# Repeated greater occipital nerve injections with corticosteroids in medically intractable chronic cluster headache: a retrospective study

**DOI:** 10.1007/s10072-021-05399-5

**Published:** 2021-06-22

**Authors:** Roemer B. Brandt, Patty G. G. Doesborg, Roy Meilof, Ilse F. de Coo, Eveline Bartels, Michel D. Ferrari, Rolf Fronczek

**Affiliations:** 1 Department of Neurology, Leiden University Medical Center, Albinusdreef 2, 2333 ZA Leiden, Netherlands; 2 Department of Anaesthesiology, Leiden University Medical Center, Leiden, Netherlands

**Keywords:** Neuromodulation, Prophylactic treatment, Pain, Nerve block

## Abstract

**Introduction:**

Current prophylactic drugs for cluster headache are associated with limited efficacy, serious side effects and poor tolerability. Greater occipital nerve injection (GON-injection) has been proven effective and safe as a single, one-time injection in episodic (ECH), and to a lesser extent, chronic cluster headache (CCH). We aim to analyse the effectiveness and safety of repeated GON-injections in medically intractable chronic cluster headache (MICCH).

**Methods:**

Clinical data of all cluster headache patients who had received at least one GON-injection between 2014 and 2018 in our tertiary headache centre were retrieved from patients’ medical records. Clinical history was taken as part of routine care shortly before and 6 weeks after GON-injection.

**Results:**

We identified 47 MICCH patients (79 injections), and compared results with 22 non-MI CCH patients (30 injections) and 50 ECH patients (63 injections). Nineteen MICCH patients received repeated injections (32 in total, range 2–8). Rates of clinical relevant improvement to a first injection were similar in all groups (MICCH: 60%, non-MICCH 73%, ECH 76%; attack freedom: MICCH: 30%, non-MICCH 32%, ECH 43%). Furthermore, no difference in response to the first and second injection was shown between groups (all *p* > 0.29). Median effect duration in MICCH was 6 weeks (IQR 2.8–12 weeks). Side effects were only mild and local.

**Conclusion:**

In this retrospective analysis, first and repeated GON-injections were well-tolerated and equally effective in MICCH as in non-MICCH, and ECH.

## Introduction

Current prophylactic drugs in the treatment of cluster headache, such as verapamil and lithium are associated with limited efficacy and poor tolerability. Chronic cluster headache (CCH) patients are defined as medically intractable (MICCH) if they experience at least three attacks per week despite consecutive prophylactic treatment trials with at least three adequate preventive treatments (amongst others: lithium, verapamil, topiramate, long-acting triptans) in the highest tolerated dose [1].

Greater occipital nerve (GON) infiltration with corticosteroids (‘GON-injection’) has been shown to be efficacious in ECH in multiple observational studies and two double-blind, placebo-controlled trials, with only mild, local side effects [2]. Since beneficial effects appear to last weeks to months, a one-time GON-injection or a single treatment cycle (from here on referred to as single GON-injection) seems mostly suitable for episodic cluster headache and has therefore been sparsely described in a well-documented group of MICCH patients [3]. Furthermore, consensus on injection compound, volume, number of injections in a single treatment cycle, and, in the case of repeated treatments, injection interval, has not yet been reached [2]. As such, (repeated) GON-injection has not yet found its place in current (inter)national treatment protocols for MICCH and is often only used as a last-resort treatment in a very limited number of headache centres in a trial-and-error approach.

This retrospective, observational study aims to assess the effectiveness, tolerability, and timing of repeated GON-injections in MICCH, compared with non-medically intractable CCH (non-MICCH) and episodic cluster headache (ECH).

## Methods

Medical records of all cluster headache patients who had received at least one GON-injection at Leiden University Medical Center (LUMC) between July 2012 and October 2018 were retrieved and analysed. Patient histories were taken directly before and 6 weeks after GON-injection as part of standard clinical care. For patients receiving repeated injections, available data on effect duration of the previous injection was incorporated in the database. Cluster headache diagnosis and MICCH classification was according to the EHF consensus statement and ICHD-3-beta criteria [1, 4]. Outcome was self-reported and classified as either ‘clinically relevant improvement’ or ‘no effect or worsening’. ‘Clinically relevant improvement’ was subdivided in ‘complete remission’ and ‘partial remission’, in which partial remission was defined as any relevant perceived improvement reported by the patient. Patients were included if self-reported outcome was registered even if data for other outcome variables was missing (e.g. data on effect duration). Ethical consideration to use the retrospective pseudonymised patient data in this study *(ID:G17.028)* was obtained from the local ethics committee (*Commissie Medische Ethiek LUMC Leiden*), the result of which was the conclusion that the proposed work does not present any ethical concerns and is of extremely low risk and no explicit informed consent was necessary.

### GON-Injection

GON-injection was performed by specialised LUMC pain anaesthesiologists. Since GON-injections were administered as part of standard clinical care, ECH patients were only injected *within* their cluster period. If spontaneous remission occurred between referral and the planned injection, patients were not injected. A 3-ml mixture of 2% lidocaine and 80 mg methylprednisolone was injected at one-third of the distance between the occipital protuberance and the mastoid process ipsilateral to the headache, directly below the superior nuchal line [5].

### Statistics

Descriptive statistics were used to report median, mean, interquartile range and standard deviations. Data were analysed for the total group of injections. Separate analysis on efficacy was performed for the first and repeated injections. Subjective improvement in subgroups was compared using a Chi-squared test or a Fisher’s exact test. Two-tailed *P*-values were deemed significant if *P* < 0.05.

## Results

### Baseline characteristics

In 47 MICCH patients, 79 injections were administered. Twenty-two non-MICCH patients received 30 injections. Forty-nine ECH patients received a total of 58 injections; 9 ECH patients received a repeated injection in a new cluster period. Baseline characteristics are shown in Table 1.

**Table 1:** Baseline characteristics and effect duration

	MICCH	Comparison groups	
		Non-MICCH	ECH
Number of patients	47	22	49
Number of injections First injections Repeated injections (total)	79 47 32	30 21 9	58 49 9
Male (*n*, %)	31 (63%)	12 (55%)	32 (65%)
Age (mean ± SD)	46 ± 14	44 ± 15	43 ± 14
Weekly attack frequency (median, IQR)	19 (13 to 35)^b^	18 (12 to 28)^c^	25 (12 to 38])^d^
Prophylactic medication^a^ (*n*, %)			
None	20 (41%)	7 (32%)	27 (55%)
At least one of the following	27 (59%)	15 (68%)	22 (45%)
Verapamil	19 (39%)	17 (46%)	15 (31%)
Lithium	5 (10%)	3 (14%)	3 (6%)
Topiramate	6 (12%)	6 (27%)	4 (8%)
Frovatriptan	6 (12%)	0	0
Pizotifen	2 (4%)	0	1 (2%)
Prednisone	0	0	2 (4%)
Acute medication^e^ *(n, %)*			
Sumatriptan s.c Oxygen	40 (85%) 35 (75%)	19 (86%) 13 (59%)	42 (86%) 29 (59%)
Effect duration in responders (weeks)			
First injection (median, IQR) Repeated injection (median, IQR)	6 (2.75 to 12) 6 (2.5 to 9)	3.5 [1 to 5] 8.5 [8 to 10.75]	6 (3.25 to 12.25) 7 (1.5 to 9.5)
Time to effect (weeks; median, IQR)			
First injection (median, IQR) Repeated injection (median, IQR)	< 1 (0) < 1 (0)	< 1 (0) 1 (1 to 2)	< 1 (0 to 1) < 1 (0)

*SD*, standard deviation; *IQR*, interquartile range; *s.c.,*subcutaneous. ^a^9 MICCH patients and 4 non-MICCH patients used more than one prophylactic drug; ^b^Data available in 46/49 patients; ^c^Data available in 21/22 patients; ^d^Data available in 37/49 patients; ^e^31 MICCH patients, 13 non-MICCH patients and 26 ECH patients used sumatriptan and oxygen

### Efficacy

In MICCH patients 28/47 (60%) of first injections, 13/19 (68%) of second injections and 7/9 (78%) of third injections resulted in a clinically relevant improvement (Table 2). Two patients received four injections and one patient received five injections with a clinically relevant improvement in all injections. No effect of sex on response was observed in the ECH (*p* = 0.296) and CCH patient group (MICCH *p* = 0.365; non-MICCH *p* = 0.178).

**Table 2:** Number of patients per injection, percentage responders and adverse events

	MICCH	Comparison groups	
		Non-MICCH	ECH
First injection (*n*) Clin. relevant improvement (*n*, %) Attack freedom (*n*, %) Adverse events^a^ (*n*, %)	47 28 (60%) 14 (30%) 15 (31%)	22 16 (73%) 7 (32%) 5 (23%)	49 37 (76%) 21 (43%) 11 (22%)
Second injection (*n*) Clin. relevant improvement (*n*, %) Attack freedom (*n*, %) Adverse events (*n*, %)	19 13 (68%) 3 (16%) 5 (25%)	3 2 (67%) - 1 (33%)	9 8 (89%) 5 (56%) 2 (22%)
Third injection (*n*) Clin. relevant improvement (*n*, %) Attack freedom (*n*, %) Adverse events (*n*, %)	9 7 (78%) 2 (22%) 1 (10%)	2 2 (100%) 1 (50%) 1 (50%)	-
Fourth injection (*n*) Clin. relevant improvement (*n*, %) Attack freedom (*n*, %) Adverse events (*n*, %)	3 2 (67%) 1 (33%) 2 (67%)	1 1 (100%) - -	-
Fifth injection (*n*) Clin. relevant improvement (*n*, %) Attack freedom (*n*,%) Adverse events (*n*, %)	2 1 (50%) 1 (50%) 1 (50%)	1 1 (100%) - -	-
Sixth–eighth injection^b^ (*n*) Clin. relevant improvement (*n*, %) Adverse events (*n*, %)	1 1 (50%) 1 (50%)	- - -	-

^a^Only minor adverse events occurred; ^b^only one patient received up to eight injections

In non-MICHH, the first injection resulted in a clinically relevant improvement in 16/22 (73%) patients with a similar response in patients receiving a second injection (Table 2). Two patients received three and eight injections respectively, with a clinically relevant improvement in all injections.

No difference in response rate between ECH and CCH patients after first injection (difference = 12%; *P* = 0.18) and second injection (difference = 21%; *P* = 0.38) was observed. Furthermore, no difference in response rates between non-MICCH and MICCH was observed after the first injection (difference = 13%; P = 0.42).

### Adverse events

Patients reported adverse events after 31/117-first injections (26%), 8/30-s injections (26%), 2/11-third injections (18%) and 4/8 for 4–8 injections (50%). Adverse events were injection site pain or discomfort (7%, *n* = 11), dysesthesia at the back of the head (5%, *n* = 9), an increase in attack frequency or severity (4%, *n* = 6), neck pain (2%, *n* = 4), side switch of cluster headache (2%, *n* = 3), other headache (1%, *n* = 2), and other (6%, *n* = 10). No SAEs were reported.

#### Effect duration and injection interval

The median effect duration in responders was 6 weeks (interquartile range (IQR) 2,63 to 9,38; 14/38 no data) in ECH and 7 weeks (IQR 3.75 to 10.25; 14/38 no data) in CCH (for specifics for each subgroup, see Table 1). Median injection interval in CCH was 100 days (IQR 86 to 131).

## Discussion

Patients with MICCH reported a clinically relevant improvement after 69% of first GON-injections, 68% of second injections and 82% of third injections. These results are in line with other studies reporting response rates in mixed groups (ECH and CCH) between 42 and 96% [2, 3, 5–12].

In contrast to these previous studies however, we focused primarily on patients with MICCH (*n* = 47). Interestingly, our MICCH patients showed a similar response to GON-injection as patients with ECH and non-MICCH patients. Although GON-injection is described as a transitional treatment in current guidelines, three earlier studies reported possible effect of repeated GON-injection in CCH with a similar percentage of patients reporting improvement [3, 7, 9]. Data could not be pooled with our data since a different injection protocol was used. However, these combined observations suggest a possible role of repeated GON-injection in the treatment of both MICCH and non-MICCH when patients experience an increase in attack frequency or severity.

Six patients reported worsening of their cluster headache after receiving the GON-injection, which could be an unwanted side effect, or, more likely, due to natural fluctuations of the disease severity.

The length of the data collection period in this study is, first of all, a reflection of the rarity of CCH and MICCH and highlights one of the challenges CCH studies face. Secondly, back in 2012, GON-injection was a relatively new treatment and the initial scarcity of evidence, especially in CCH, resulted in an initial low referral rate for GON-injection. However, because of the positive clinical results and emerging evidence, (repeated) GON-injections are being administered more frequently, although incorporation in the general treatment guidelines requires further robust prospective trials.

### Adverse events

No serious adverse events occurred after GON-injection. The most frequently occurring adverse event was mild pain at the injection site. No systemic side effects were reported, which is in line with our expectations since the combined total dose of corticosteroids after multiple injections is relatively low when compared with an oral prednisone regimen. However, treating physicians should be alert on side effects and treatment should be halted when signs of local alopecia, subcutaneous atrophy or corticosteroid toxicity develop. Because of the accumulative local corticosteroid dosage in repeated injections, a lower corticosteroid dose per injection could be preferable. However, to date, no dose response studies have been conducted so no definitive dose recommendation can be given but the possibility of lower corticosteroid dosages should be further explored in prospective trials.

### Mechanism of action

The mechanism of action is not understood. It has been suggested that the beneficial effect is due to a systemic effect of the injected corticosteroids [6]. However, the dosage of systemic corticosteroids is much higher than a single injection with 80 mg methylprednisolone. Furthermore, attacks have been known to re-occur when tapering oral corticosteroids, while the effect of GON-injections can last weeks to months, deeming a systemic effect not likely. It has been shown that the GON converges with the first division of the trigeminal nerve in second-order nociceptors in the trigeminal nucleus caudalis. Functional interaction between the GON and the trigeminal nerve has been shown by an increased latency of the bilateral R2 components of the blink reflex after ipsilateral stimulation to the GON-injection [13]. Since the onset of effect is within days, a central neuromodulatory effect seems unlikely. Currently, the most plausible mechanism of action is modification of trigeminal nociceptive transmission through reduced transmission in normal unmyelinated C-fibres [14]. Furthermore, since mechanism of action of different corticosteroids is presumably similar, no difference in effect between type of corticosteroid should be expected, as demonstrated by previous studies obtaining comparable results with different injection compounds [2].

### Limitations

Due to retrospective nature of this study, we were limited in the amount of details we could extract from patient histories. Because of this limitation, we were unable to obtain accurate data on attack frequency, which is the preferred outcome in studies investigating prophylactic therapy for cluster headache [15]. Furthermore, data on frequency of acute medication use, which can be seen as a relevant proxy of attack frequency and intensity, was unavailable as well. We therefore defined a ‘clinically relevant response’ as a self-reported positive effect of the GON-injection. Other studies usually define ‘clinically relevant response’ as a reduction in attack frequency of at least 50%. This definition could lead to an overestimation of partial responders in this study when compared to other studies. However, despite the subjective nature of defining a clinical relevant response, a substantial number of patients reported a clear-cut complete remission. Furthermore, due to the nature of the patient group (medically intractable chronic cluster headache patients), a subjective improvement reported by patients who have previously tried and failed other prophylactic medication implies a real-life treatment effect. However, because people with MICCH inherently have experienced multiple treatment failures, it could be argued that people with MICCH could have a lower threshold for treatment satisfaction when compared to ECH patients. This could imply that the absolute reduction in attack frequency or intensity (the objective treatment effect) could be lower in the MICCH group when compared to the ECH group, even when the ‘clinically relevant response’ (subjective treatment effect) is comparable.

Treatment evaluation was obtained 6 weeks after GON-injection. Data on effect duration longer than 6 weeks was only available for patients returning for a repeated injection. Because of this time interval, it is possible that the slightly higher percentage of episodic cluster headache patients that reports clinically relevant improvement is for a part due to spontaneous remission. However, since attack frequency in chronic cluster headache is presumably relatively stable, we do not expect a large effect of this phenomenon in the CCH and MICCH patient groups. Furthermore, in many cases, there was a clear-cut complete remission, which is normally not spontaneously expected in these patient groups*.*

Due to the retrospective design, most patients who received a repeated injection had a positive response to the first injection. Treatment response for repeated injections is therefore based mainly on patients who responded to the previous injection. However, since response rates appear to be more or less stable after repeated injection in this study and in a previous study, we hypothesise that the success chance of a GON-injection is constant despite the outcome of a previous injection [9]. Only one study has reported limited data on this, showing that the response to the first injection does not predict the response to a second injection [7]. This raises the question whether one should repeat a GON-injection, even when a previous injection was not successful. However, to date, not enough data are available to support this hypothesis. Furthermore, effect of repeated injections in combination with other prophylactic drugs should be further explored in prospective trials, using robust outcome measures such as absolute attack frequency and frequency of acute medication use.

## Conclusion

In our retrospective cohort, single GON-injections were an effective and safe prophylactic treatment for MICCH, similar to non-MICCH and ECH. Transient attack freedom was achieved in one-third of injections in all groups. Repeated injections were well-tolerated and showed similar effectiveness. Further data is needed to establish the optimal interval between repeated GON injections and long-term efficacy and safety. Our retrospective data suggests an emerging role for repeated GON-injection in the treatment of medically intractable chronic cluster headache.

## Data Availability

The datasets generated during and/or analysed during the current study are available from the corresponding author on reasonable request.

## Declarations

### Clinical implications

• GON-injection can be a viable treatment option in medically intractable chronic cluster headache.

• Repeated GON-injections show similar effect to a first GON-injection in (medically intractable) chronic cluster headache.
